# High Content Analysis of Primary Macrophages Hosting Proliferating *Leishmania* Amastigotes: Application to Anti-leishmanial Drug Discovery

**DOI:** 10.1371/journal.pntd.0002154

**Published:** 2013-04-04

**Authors:** Nathalie Aulner, Anne Danckaert, Eline Rouault-Hardoin, Julie Desrivot, Olivier Helynck, Pierre-Henri Commere, Hélène Munier-Lehmann, Gerald F. Späth, Spencer L. Shorte, Geneviève Milon, Eric Prina

**Affiliations:** 1 Institut Pasteur, Imagopole, Paris, France; 2 Institut Pasteur, Laboratoire Immunophysiologie et Parasitisme, Département de Parasitologie et Mycologie, Paris, France; 3 Institut Pasteur, Unité Chimie et Biocatalyse, Département de Biologie Structurale et Chimie, Paris, France; 4 CNRS, UMR 3523, Paris, France; 5 Institut Pasteur, Unité Parasitologie Moléculaire et Signalisation, Département de Parasitologie et Mycologie, Paris, France; 6 CNRS URA 2581, Paris, France; The Ohio State University, United States of America

## Abstract

**Background/Objectives:**

Human leishmaniases are parasitic diseases causing severe morbidity and mortality. No vaccine is available and numerous factors limit the use of current therapies. There is thus an urgent need for innovative initiatives to identify new chemotypes displaying selective activity against intracellular *Leishmania* amastigotes that develop and proliferate inside macrophages, thereby causing the pathology of leishmaniasis.

**Methodology/Principal Findings:**

We have developed a biologically sound High Content Analysis assay, based on the use of homogeneous populations of primary mouse macrophages hosting *Leishmania amazonensis* amastigotes. In contrast to classical promastigote-based screens, our assay more closely mimics the environment where intracellular amastigotes are growing within acidic parasitophorous vacuoles of their host cells. This multi-parametric assay provides quantitative data that accurately monitors the parasitic load of amastigotes-hosting macrophage cultures for the discovery of leishmanicidal compounds, but also their potential toxic effect on host macrophages. We validated our approach by using a small set of compounds of leishmanicidal drugs and recently published chemical entities. Based on their intramacrophagic leishmanicidal activity and their toxicity against host cells, compounds were classified as irrelevant or relevant for entering the next step in the drug discovery pipeline.

**Conclusions/Significance:**

Our assay represents a new screening platform that overcomes several limitations in anti-leishmanial drug discovery. First, the ability to detect toxicity on primary macrophages allows for discovery of compounds able to cross the membranes of macrophage, vacuole and amastigote, thereby accelerating the hit to lead development process for compounds selectively targeting intracellular parasites. Second, our assay allows discovery of anti-leishmanials that interfere with biological functions of the macrophage required for parasite development and growth, such as organelle trafficking/acidification or production of microbicidal effectors. These data thus validate a novel phenotypic screening assay using virulent *Leishmania* amastigotes growing inside primary macrophage to identify new chemical entities with *bona fide* drug potential.

## Author Summary

Leishmaniases are neglected diseases caused by protozoan parasites that belong to the genus *Leishmania*. No vaccine exists against any form of leishmaniasis and most of the existing anti-leishmanial drugs have serious side effects. Current strategies for discovering new leishmanicidal molecules are largely using inappropriate types of host cells or employing the irrelevant insect-specific parasite stage. We presented here an innovative and biologically relevant drug screening assay based on the use of the mammalian macrophage host cells and *Leishmania* pathogenic amastigotes. Our visual fluorescence assay has been established with the objective to screen diverse small-molecules including chemicals and natural compounds that selectively target intra-macrophagic amastigotes without displaying toxicity for the macrophage host cells. The validation of a miniaturized assay, relying on automated handling of biological materials and fluorescent imaging probes, image acquisition, data storage and analysis is presented. Based on robust statistical methods and quality control metrics, the data analysis pipeline allows for the classification of compounds based on their effect on parasite and macrophage survival observed after three days of treatment.

## Introduction

Human leishmaniases are diseases that are endemic throughout tropical and subtropical areas causing severe morbidity and mortality with an estimated worldwide incidence of 1,500,000 newly reported clinical cases per year. More than 12 million people are currently infected and 350 million people are at risk [1].

Causal agents of human leishmaniases are Euglenozoan digenetic parasites belonging to the Trypanosomatidae family and the genus *Leishmania*. They are cycling between i) blood-feeding phlebotomine insects where they develop as extracellular flagellated promastigotes, and ii) a range of mammals, including rodents, canids and human where they develop as obligatory intracellular amastigotes. Depending on both the *Leishmania* species and the mammal host genetic and immune status, four main clinical forms of leishmaniasis can be distinguished, including i) the visceral form, which causes long-term fever, weight loss, hepatosplenomegaly and pancytopenia, and is usually fatal if left untreated, ii) the cutaneous form characterized by single or multiple lesions, which generally self-cures over months, iii) the muco-cutaneous form affecting mainly nasopharyngeal mucosa characterized by extensive tissue destruction causing severe facial disfiguration and respiratory disturbances, and iv) a diffuse form in which non-ulcerating lesions are spread over large skin areas. Leishmaniases result in a strong social stigma and marginalization for infected individuals and has an important negative impact on welfare and productivity of people from developing countries [2]–[4].

The current treatment of leishmaniasis essentially relies on chemotherapy as, to date, neither preventive nor therapeutic vaccines are available. Pentavalent antimonials have been used for more than 70 years and are still the recommended first line of treatment. However, these drugs not only require a long course of parenteral treatment with repeated injections and careful monitoring in health centers, but also display variable efficacy and toxic side effects [1], [5]–[7]. Alternative therapeutic approaches based on the use of Amphotericin B (AmphoB), an antifungal polyene antibiotic, and its lipid-carrier formulations have been successfully applied when first line drugs were no longer effective [8], [9]. Nevertheless, the very high costs of these drugs limit their widespread use. In recent years, new molecules such as the anti-neoplastic agent Miltefosine, new treatments such as drug combinations, or new administration routes like topical formulations and oral administration, have been introduced in anti-leishmanial chemotherapy. However, in view of the recent spreading of *Leishmania* showing resistance to the limited number of existing drugs in various parts of the world, there is an urgent need to develop new, safe, fast acting, and affordable treatments.

While several new chemical scaffolds have been identified recently, they were issued from screening campaigns that were primarily based on the use of extracellular promastigotes [10], [11], *i.e.* the developmental stage that differentiates within the mid gut of sand fly hosts. It is noteworthy that promastigotes differ significantly from amastigotes with respect to morphology, the composition of their surface glycocalyx, and metabolism. These biological differences reflect distinct developmental programs that adapt *Leishmania* for extra- and intracellular survival in the phlebotomine mid gut and the macrophage parasitophorous vacuole (PV), respectively [12]–[16]. Conceivably, this stage specific biology has important consequences on how the parasite responds to chemicals [17]–[19]. When comparing the leishmanicidal activity of antimony in infected animals versus cultured parasites, the antimony susceptibility determined *in vivo* correlated better with the *in vitro* assays performed with intracellular amastigotes than with extracellular promastigotes [20]. Moreover, host features such as the permeability of plasma- and PV- membranes or the presence of molecules able to directly interfere with or metabolize chemicals are key parameters to consider for discovering compounds with selective activity against the intracellular amastigote stage. *Leishmania* survival within macrophages depends on the capacity of amastigotes to evade or to resist the innate host cytotoxic activities. It is possible that rescue of these activities will lead to efficient intracellular amastigote destruction. Thus, an assay based on the phenotype classification of primary macrophages hosting *Leishmania* amastigotes may allow to discover compounds that confer to infected macrophage full leishmanicidal activities [18], [21]–[24]. With the aim to establish a biologically relevant screening system that accounts for all of these considerations, we designed and validated a miniaturized High Content Analysis assay, relying on primary mouse macrophages hosting virulent *L. amazonensis* amastigotes. This approach overcomes limitations associated with promastigote-based screens [19], [25]. It allows the rapid selection of compounds that are able to interfere with *Leishmania* amastigote growth and survival within primary macrophages either directly, or indirectly by modifying macrophage organelle trafficking or acidification required for intracellular parasite growth. Based on robust statistical methods, quality control metrics, hit identification classification and validation, we developed a powerful data analysis pipeline that provides for each tested compound metrics on amastigote load and their toxic effect on host macrophages at the single cell level and for the entire sample population analyzed.

## Materials and Methods

### Experimental procedures and methodologies

#### Ethics statement

All animals were housed in our A3 animal facilities in compliance with the guidelines of the A3 animal facilities at the Pasteur Institute which is a member of Committee 1 of the “Comité d'Ethique pour l'Expérimentation Animale” (CEEA) - Ile de France. Animal housing conditions and the protocols used in the work described herein were approved by the “Direction des Transports et de la Protection du Public, Sous-Direction de la Protection Sanitaire et de l'Environnement, Police Sanitaire des Animaux under number B75-15-27 in accordance with the Ethics Charter of animal experimentation that includes appropriate procedures to minimize pain and animal suffering. EP is authorized to perform experiment on vertebrate animals (license 75–1265) issued by the “Direction Départementale de la Protection des Populations de Paris” and is responsible for all the experiments conducted personally or under his supervision as governed by the laws and regulations relating to the protection of animals.

### Production of macrophages

Female Swiss nu/nu and BALB/c mice, between 8- and 12-week of age, were obtained from Charles River. Bone marrow cell suspensions recovered from tibias and femurs of BALB/c mice were suspended in DMEM medium (Gibco, life technologies) containing 4 g/L glucose, 1 mM pyruvate and 3.97 mM L-Alanyl-L-Glutamine, 10% heat-inactivated fetal calf serum (FCS, Dominique Dutscher SAS), streptomycin (50 µg/mL) and penicillin (50 IU/mL) (Biochrom AG, IBS International) (culture medium) and with 50 ng/mL recombinant mouse CSF-1 (rmCSF-1) (ImmunoTools). Cells were distributed in bacteriologic plastic flasks (Corning Life Science, 7×10^5^ cells/ml) and were incubated at 37°C in a 7.5% CO_2_ air atmosphere for 6 days.

### FACS analysis of mouse macrophage-restricted markers

Six days old bone marrow-derived, loosely adherent macrophages were washed with Dulbecco's phosphate buffered solution (PBS) and detached by gentle flushing (25 min at 37°C) with pre-warmed 1% EDTA in PBS without Ca^2+^ and Mg^2+^ (Biochrom AG). Recovered macrophages were suspended in either culture medium for HCS assay or cold PBS with 2% FCS and 0.05% sodium azide (PBS-FCS-Az) for FACS quality controls. Macrophages for FACS analysis were transferred to round-bottomed 96-well plates (Corning Costar) at a concentration of 3×10^5^ cells/well. All subsequent steps were performed on ice and with ice-cold reagents. Cells were centrifuged (300 *g*) for 5 minutes and then incubated in PBS-FCS-Az supplemented with 10% donkey serum for 20 minutes. After centrifugation, cells were incubated for 30 minutes in PBS-FCS-Az containing a combination of fluorescent reporter-conjugated antibodies. Flow cytometry results were acquired on a Gallios flow cytometer (Beckman Coulter) and data analyzed with the Kaluza software package (Beckman Coulter).

The anti-mouse mAbs were purchased from Pharmingen for FITC-labeled 2G9 anti I-A^d^/I-E^d^ clone, or eBioscience for the followings clones: e450-conjugated N418 anti-CD11c (p150/90), APC-conjugated M1/70 anti-CD11b/CR3 α-chain, PE-conjugated 16-10A1 anti-CD80/B7-1, APC-conjugated BM8 anti-F4/80, and PE-conjugated AFS98 anti-CD115.

### Sampling and preparation of *Ds*Red 2 *L. amazonensis* amastigotes

*L*. *amazonensis* strain LV79 (MPRO/BR/1972/M1841) was genetically modified by chromosomal integration of the fluorescent *Ds*Red2 molecule [26] and propagated in Swiss nu/nu mice by subcutaneous injection of 10^6^ amastigotes into hind footpad. Six to eight weeks after amastigote inoculation lesions were excised and amastigotes purified by a modified version of the method originally described by M. Rabinovitch and colleagues [27]. Briefly, lesions were minced in PBS supplemented with streptomycin (100 µg/mL) and penicillin (100 IU/mL), and disrupted by hand in a glass homogenizer. Tissue debris were removed by 2 rounds of centrifugation at 30 *g* for 10 mn at 4°C. Amastigotes present in the supernatant were washed 2 times by centrifugation at 1500 *g* for 10 mn at 4°C before distribution in macrophage cultures. A high number of live amastigotes expressing homogenous levels of *Ds*Red2 were recovered as determined by FACS analysis (data not shown).

### Chemicals

Reference compounds, Leucine Methyl Ester (Leu-OMe) [27], AmphoB and cycloheximide, were solubilized in DMSO (Sigma-Aldrich). Based on literature data, we selected 60 compounds with established or potential leishmanicidal, anti-fungal or anti-microbial and cytotoxic activities to validate our experimental and data analysis pipelines. Details about their origins, working concentrations and known activity are provided in Table S1. We defined the following control conditions: **C−** 1% DMSO-, **C+**, 0.5 µM AmphoB- and **C†** 180 µM cycloheximide. All compounds were assayed at 10 µM or as stated in Table S1. The detailed plate maps used in this study with the position of controls are depicted in Tables S2 and S3 (see table legend for details).

### Assay preparation

Six days-old bone marrow-derived adherent macrophages were recovered as described above and deposited in culture-treated flat-optically clear bottom black 384-well plates (CellCarrier plate, PerkinElmer) at a density of 1.5×10^4^ cells in 70 µl of medium supplemented with 12 ng/ml of rmCSF-1 per well, resulting in a 80% confluence monolayer without formation of cellular aggregates. Five hours later, purified *Ds*Red2-expressing amastigotes were added to the macrophages at a multiplicity of 3 parasites per host cell (MOI = 3) (30 µl/well). Macrophage cultures were further incubated at 34°C, which is the permissive temperature for the surviving and multiplication of LV79 amastigotes [28], [29]. After an overnight incubation period, more than 85% of macrophages harbored intracellular parasites that were already multiplying in growing PVs [30]. At this time, compounds (Tables S2, S3) were added to macrophages (1 µl/well) resulting in a final concentration of 1% DMSO in each well. The cultures were then maintained 3 days at 34°C until processing for image acquisition.

One hour before image acquisition, the cells were incubated with vital cell-permeant dyes Hoechst 33342 (12 µM) and LysoTracker DND-26 (1 µM) (life technologies). Optimization of operating parameters included an automated dispensing of biological material (macrophages and amastigotes) and chemicals (vehicle, compounds) using the Te-MO 96-channel pipetting head of a TECAN Freedom EVOware platform located under laminar flow in a BSL2 facility. The homogeneity and the reproducibility of all pipetting procedures were assessed by quantitative image analysis during assay development (data not shown).

### Image acquisition and analysis

After 60 minutes of contact with fluorescent reporters, three channel images were acquired in a fully automated and unbiased manner using a spinning disk confocal microscope (OPERA QEHS, PerkinElmer Technologies) and a 10× air objective (NA = 0.4) using the following sequential acquisition settings: (i) 561 nm laser line excitation, filter 600/40 for *Ds*Red2 detection, (ii) 488 nm laser line excitation, filter 540/75 for Lysotracker DND-26 detection and (iii) 405 nm laser line excitation, filter 450/50 for Hoechst 33342 detection. Fifteen images per channel, covering the entire surface of each well, were collected for reliable statistical analysis taking into account potential cell-distribution and spatial compound effect biases.

The images were transferred to the Columbus Conductor™ Database (Perkin Elmer Technologies) for storage and further analysis. The image analysis was performed by batches in Columbus using custom designed image analysis scripts developed beforehand with the Acapella Image analysis software (version 2.5 - Perkin Elmer Technologies). The script was subdivided in three object segmentation subroutines detecting successively and independently the nuclei, the PV and the Am with their respective associated features (number, size, and intensity); the living macrophage population characterisation is based on a combination of host cell nucleus size and intensity features which are key characteristics of the relative fitness of the macrophage population. Finally, all the quantitative data generated were exported in readable file format to be subsequently analyzed in the data analysis workflow described below.

### Data analysis workflow

To validate our pipeline, selections of image analysis outputs, including macrophage nuclei, PV and Am counts that best represented typical compound-induced phenotypes, were used. We applied a standardized data analysis workflow to automate and validate the interpretation of the large amount of data generated by image acquisition and analysis; it consists of the following three main classical hands-off steps [31]:

1. **Data normalization.** A robust version of the Strictly Standardized Mean Difference (SSMD*), based on median difference and median absolute deviation (MAD), was used for normalization of the samples which effectively minimizes the effect of outliers [32].
2. **Quality control.** The Quality Control (QC) assessment of the assay is essentially derived from the estimation of the magnitude of difference between control populations. A combination of two common QC metrics, the robust Z′factor [33] and the SSMD [34] were used as criteria for plate data rejection or acceptance. Because of the strong induced phenotypes of our controls population, we created a new intermediary threshold for the Z′factor (“good”) (Table S4).
3. **Hit definition.** We used a sequential approach to classify the data sets based on their degree of significance (SSMD* values) for the chosen output parameters. To this end, based on 3 original readouts (macrophage, amastigote and PV counts) and different variables derived from these counts i.e. Total Macrophages (TM), Healthy Macrophages (HM), Viability Index (VI) for the HM to TM ratio and PV to HM ratio (PV/HM), the following steps were implemented (Figure S1): (**Step 1**) fitness of the host cell population with the VI parameter, (**Step 2**) fine tuning of the health status of the macrophage population by dichotomous classification of the **TM** and **HM** parameters, and (**Step 3**) leishmanicidal effect by dual classification of the **Am** and **PV/HM** parameters. We directly classified the **Am** output in the specific case where the effects on the macrophage population were significantly different of the control population (high toxicity).

## Results/Discussion

### The existence of giant *L. amazonensis*-containing vacuoles is a reliable indicator for the presence of intracellular proliferating amastigotes

**Figure 1:**
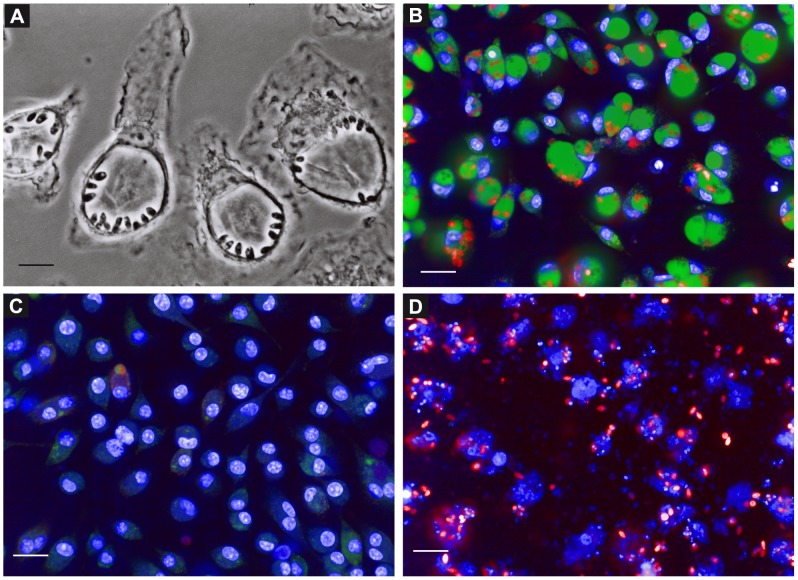
*Leishmania amazonensis* lodge and multiply within giant acidic vacuoles. Six days-old macrophages were seeded into 384-well microplates and incubated with *L. amazonensis Ds*Red2 expressing amastigotes for 48 h. (**A**) Phase contrast image demonstrating the lodging of the amastigotes within giant vacuoles. Images of living cells were acquired with a 100× immersion objective on a Zeiss Axiovert 200 M microscope. Scale bar corresponds to 5 µm. (**B–D**) Images of living infected macrophage cultures incubated 60 min with fluorescent reporters were acquired on a confocal plate reader (Opera QEHS) after 3 days of incubation with the compounds. 20× confocal images acquired from samples treated with (**B**) 1% DMSO (C-), (**C**) 0.5 µM Amphotericin B (C+) and (**D**) 180 µM Cycloheximide (**C†**) are depicted. Scale bars represent 20 µm. Macrophage nuclei and PV were stained with Hoechst 33342 (Blue) and LysoTracker DND-26 (Green), respectively. Amastigotes expressing *Ds*Red2 are represented in red.

Current screening protocols for the discovery of anti-leishmanial compounds are compromised by both the types of parasites and host cells employed. First, the use of culture-derived promastigotes and axenic amastigotes are not reflecting the biology and environment of parasites inside the macrophage. In addition Pescher and colleagues recently demonstrated that axenic amastigotes were not able to induce acute visceral disease in hamsters compared to tissue-derived amastigotes thus minimizing the potential interest of using host-free parasites [35]. Second, the use of macrophage cell lines as host cells for *Leishmania* is problematic due to the mandatory use of chemicals to induce terminal macrophage differentiation, chemicals that are known to result in modulation of macrophage sensitivity to compounds, thus compromising the interpretation of screening results [18], [36], [37]. Finally, the combination of both *i.e.* using host cell lines infected with culture-derived parasites results in i) the presence of large quantity of extracellular proliferating promastigotes and ii) a lower rate of host cells hosting metacyclic promastigotes able to differentiate into cell-cycling amastigotes. To overcome these limitations, we set up a reliable assay based on the use of mouse primary macrophages and highly virulent lesions-derived amastigotes of *L. amazonensis*, which were genetically modified to stably express a *Ds*Red2 fluorescent reporter [26]. Homogeneous populations of macrophages differentiated from bone marrow progenitors by incubation with Colony Stimulating Factor 1 (CSF-1) [29] (Figure S2) were distributed in 384-well plates, and amastigotes freshly prepared from Swiss nude mouse lesions [38] were then added to the macrophage monolayer. In contrast to promastigote-based protocols, *L. amazonensis* amastigotes were readily phagocytized by macrophages leading to a high and sustained infection rate. After a few hours, amastigotes were already multiplying, resulting in the development of large PVs, a phenotypic hallmark of a successful intracellular *L. amazonensis* infection (Figure 1A) [30], [39]. No extracellular amastigotes can be evidenced either by phase contrast or confocal fluorescence microscopy (Figures 1A, 1B). The PV property to accumulate the cell-permeant LysoTracker DND-26, a fluorescent weak base probe, is a signature of the sustained fusion of macrophage late endocytic organelles with amastigotes-containing phagosomes (Figure 1B) [39], [40]. When amastigote growth is blocked or amastigotes are killed in presence of a leishmanicidal agent like AmphoB, PVs are either strongly reduced in size or no longer detected (Figure 1C). The disappearance of PVs was correlated in a previous study to elimination of intracellular amastigotes as shown by fluorescence and differential interference contrast microscopy, and real-time quantitative PCR [29]. The presence/absence of PVs represents thus a powerful digital readout to monitor a leishmanicidal effect, a criterion we already used successfully in a non-automated visual assay for selecting 2-quinoline derivatives with activity against intracellular *L. amazonensis* amastigotes [29], [41]. Concomitantly, our assay allows monitoring the health status of host macrophages by visualizing their nuclear morphology with the permeant DNA probe Hoechst 33342. In presence of a toxic compound like cycloheximide, dead macrophages were easily differentiated from healthy cells and identified by the loss of nuclear integrity (Figure 1B, 1C, 1D). Based on this biologically relevant and quantifiable infection system we established an automated phenotypic screening pipeline described below.

### Image acquisition and analysis pipeline

**Figure 2:**
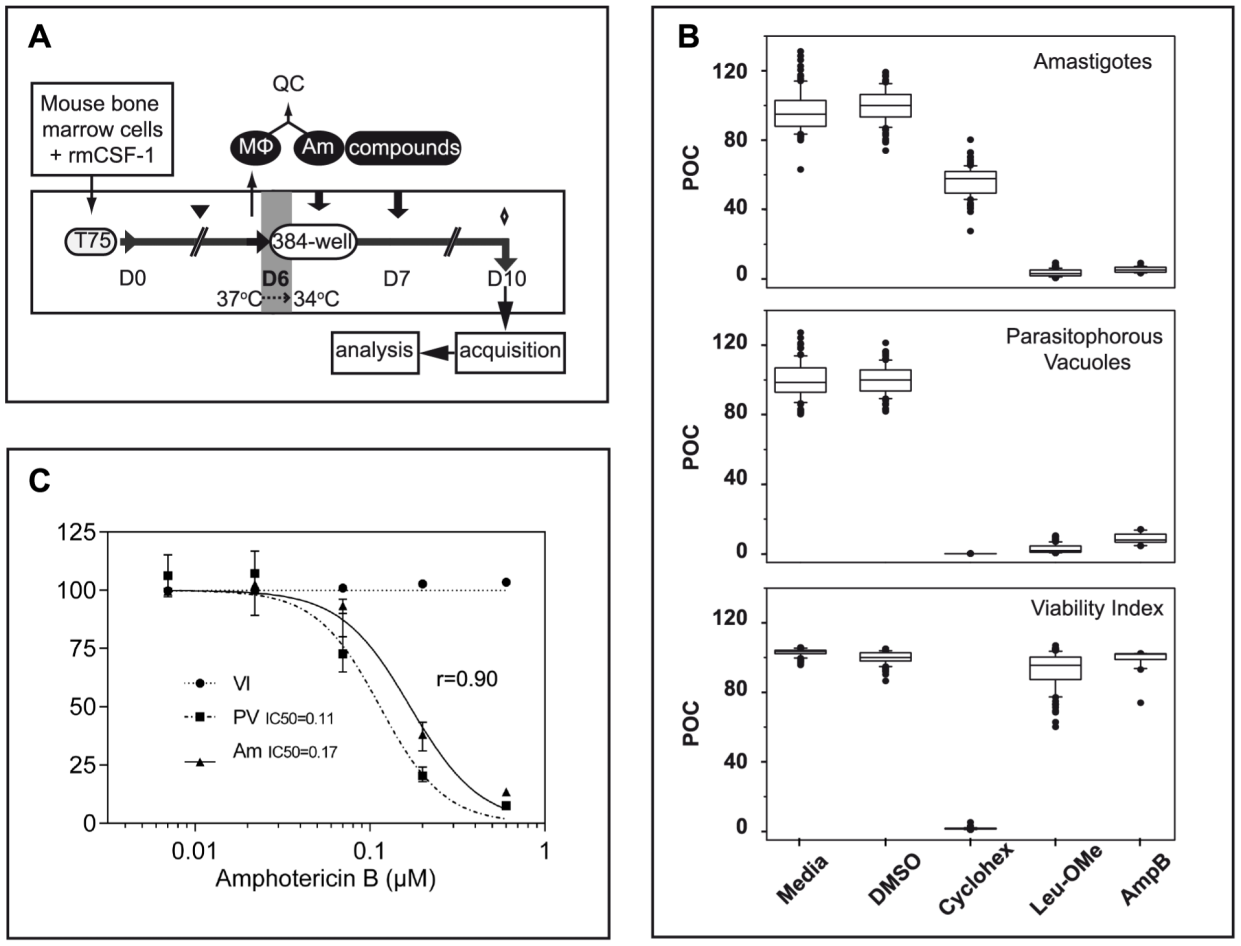
Assay Workflow chart and outputs. (**A**) Experimental pipeline. Bone marrow cells recovered from BALB/c mice were cultured in presence of 50 ng/mL rmCSF-1 in hydrophobic flasks at 37°C allowing CSF-1 responsive progenitors to further develop along the macrophage lineage. Fresh medium was added at day 3 (black triangle) and differentiated adherent macrophages (MΦ) were harvested at day 6 and seeded into 384-well culture-treated optical bottom plates in the presence of a lower concentration of rmCSF-1 to prevent cell death. Macrophage cultures were then maintained at 34°C until the end of the experiment. Amastigotes (Am) freshly isolated from nude mouse footpads were added (ratio of 3 amastigotes per macrophage) to macrophages 5 hours after macrophages plating. Compounds are added 19 hours later for a contact period of 3 days. One hour before image acquisition on the confocal plate reader, the fluorescent reporters Hoechst 33342 and LysoTracker DND-26 were added to the macrophage cultures (diamond). All dispensing procedures in the 384-well plates were performed using an automated liquid handling robot without any washing step. (**B**) Boxplots with whiskers from minimum to maximum of Percent Of negative Control (POC) for the amastigote (Am), parasitophorous vacuoles (PV) and Viability Index (VI) after the following treatments: no compound added (Media), 1% DMSO, 180 µM cycloheximide, 2 mM Leu-oMe and 1 µM Amphothericin B (AmpB) leishmanicidal drug. Sets of 96 data points for each are issued from a representative validation 384-well plate. (**C**) AmphoB dose-response curves were expressed as POC for (VI) (black circle), (PV) (black square) and (Am) (black triangle). Each concentration has been tested in quadruplicates; error bars represent the data range. IC50 curve fitting and the Spearman correlation coefficient (r, for n = 5) between the IC50 dose curves for the PV and Am outputs were calculated using GraphPad Prism 6 software (GraphPad Software Inc., San Diego, CA).

To minimize any experimentally induced biases, we developed a linear procedure that consists of the sequential addition of mouse bone marrow-derived macrophages, purified tissue-derived amastigotes and chemicals in 384-well optical imaging clear bottom plates, without any washing steps (Figure 2A). This protocol minimizes the potential heterogeneity between wells, thereby avoiding sample perturbation and artifacts over the subsequent incubation period. The procedure is finalized after 3 days of co-culture by the addition of the fluorescent reporters LysoTracker DND-26 and Hoechst 33342 one hour before image acquisition. To validate our procedure, images were acquired in numerous control wells for the 3 different fluorescent reporters corresponding to the counts of *Ds*Red2-tagged amastigotes, LysoTracker-positive PVs and macrophage Hoechst-stained nuclei (see next paragraph for a detailed description of the procedure). In order to avoid biases due to potential heterogeneity of the macrophage monolayer that could arise over time, and to increase the size of the population analyzed, image acquisitions were performed at low magnification using a dry 10× objective for the entire surface of the wells, resulting in acquisition and analysis of all cells for each sample. The images were thereafter segmented using Acapella scripts and the outputs normalized and expressed as percentages of media control (Figure 2B). The presence of 1% DMSO (C-) did not induce toxic effect on macrophages as demonstrated by the similar values obtained for the VI (Figure 2B bottom panel and Figure S3). On the contrary, the presence of a toxic compound for the macrophage, like cycloheximide, was easily evidenced by the dramatic decrease of the VI compared to untreated or DMSO-treated samples (Figure 2B, bottom panel). As expected, in presence of 2 leishmanicidal agents L-Leu-oMe and AmphoB, amastigote and PV counts were significantly reduced compared to negative controls (Figure 2B, top and middle panels) without inducing toxicity on host macrophages (Figure 2B, bottom panel). When performing a comparative analysis between amastigote and PV counts, we observed a good correlation in dose-response experiments using known leishmanicidal agents (Figure 2C and data not shown). Specifically, the IC50 of AmphoB was estimated at 0.11 µM and 0.17 µM for the PV and amastigote output, respectively, which, furthermore, are consistent with published values [10]. Because the PV readout can be efficiently quantified at lower magnification (10× objective), allowing for the analyses of the entire well per sample, we performed the subsequent screen using the presence/absence of PVs as the principal readout for parasite burden generating statistically highly relevant screening data. The box-and-whisker diagrams depicted in Figure 2B for the different variables strengthen and validate the homogeneity and the reproducibility of pipetting procedures, including the chemical distribution routine, used in this assay. The counts of macrophage nuclei, PVs, and amastigotes were subsequently used to establish a data analysis pipeline described in the following.

### Development of the data analysis pipeline

We applied our experimental approach to a small compound library combining known leishmanicidal drugs, newly published structures and antifungal or cytotoxic agents (Table S1). These compounds were initially tested in quadruplicates per plate in 2 different experiments (plates P1–P2 for Exp1 and P3–P4 for Exp2). In experiments 3 to 7, we used single data points for each compound on each plate (plates P5 to P9 corresponding to Exp3 to Exp7) at a concentration of 10 µM unless stated otherwise (Table S1). The precise design of the assay plates is described in Tables S2 and S3. In P5 to P9, we removed the “media” negative control, which was redundant to the “vehicle” control (1% DMSO) since no toxicity towards the macrophage and amastigote populations could be evidenced (Figure 2B and Figure S3). We also included in these plates cycloheximide-treated cells as positive control for macrophage toxicity (**C†**), which allowed us to more adequately classify compounds with strong toxic effect on macrophages and only very weak effect on amastigotes (Figures 1D and 2B) as shown in our assay validation plate.

We developed thereafter a stringent data analysis pipeline based on strong proven statistical methods for quality control, normalization and ranking of the results to validate and analyse the screening outcomes as described in the methods section. The three original readouts chosen from the image analysis (count of macrophages, PVs and amastigotes) are *de facto* independent and were used to define five variables: Total Macrophages **(TM)**, Healthy Macrophages **(HM)**, Viability Index **(VI = HM/TM)**, total number of Amastigotes (**Am**), and the ratio between PVs and healthy macrophages **(PV/HM)**. These variables allow for precise phenotype discrimination and efficient hit identification. Critical main hands-off steps of the data analysis pipeline are described below [31].

#### i) Quality control (QC)

For each plate, we validated not only the robustness of the controls but also the reproducibility of the assay. By combining the two typical QC metrics Robust z′-factor [33] and strictly standardized mean difference (SSMD*), we validated each plate for every variable analyzed (Table S4). The QC scores computed for all variables in every plate ranged from acceptable to excellent (Table 1).

**Table 1:** QC metrics by variables (Am, PV/HM and VI) expressed as robust Z′factor and (SSMD) values.

Plate	Am (C+C−)		PV/HM (C+C−)		VI (C†C−)	
	Z′ Factor	SSMD	Z′ Factor	SSMD	Z′ Factor	SSMD
P1	**A** (0.25)	**A** (−4.30)	**E** (0.54)	**E** (−7.90)	NA	NA
P2	**G** (0.48)	**G** (−6.32)	**G** (0.48)	**G** (−6,46)	NA	NA
P3	**G** (0.49)	**G** (−6.14)	**G** (0.38)	**G** (−6.54)	NA	NA
P4	**E** (0.58)	**E** (−7.88)	**E** (0.57)	**E** (−8.52)	NA	NA
P5	**E** (0.71)	**E** (−10.77)	**E** (0,66)	**E** (−12.54)	**E** (0.93)	**E** (−50.11)
P6	**A** (0.16)	**A** (−4.50)	**A** (0.22)	**G** (−5.38)	**E** (0.85)	**E** (−27.23)
P7	**A** (0.25)	**A** (−4.26)	**E** (0.64)	**E** (−11.88)	**E** (0.93)	**E** (−51.79)
P8	**E** (0.64)	**E** (−10.35)	**G** (0.36)	**G** (−6.27)	**E** (0.95)	**E** (−81.29)
P9	**E** (0.56)	**E** (−8.38)	**E** (0.73)	**E** (−15.03)	**E** (0.95)	**E** (−69.57)

To validate the robustness of controls in each plate, the QC metrics were performed for each variable using different controls depending on the biological readout *i.e.* leishmanicidal activity or toxicity on host cells: C− (64 and 24 wells for P1–P4 and P5–P9, respectively)/C+ (n = 20) for (Am) and (PV/HM) variables and C− (n = 24)/C† (n = 20) for (VI). Of note is the fact that the QC metrics for the VI variable was only available in the P5–P9 when the C† control was included (NA: not applicable).

#### ii) Hit definition

To automatically select and identify hits, we calculated the SSMD* for each chosen variable in each well [32]. This metrics was used on the control populations as QC indicator and also represents the magnitude of the difference between a treatment and the negative reference phenotype. Depending on these SSMD values and the category threshold for the different variables compounds were categorized using the multivariate classification methodology described below.

#### iii) Hit classification and validation

The compound-induced phenotypes were classified into categories based on their leishmanicidal activity and host macrophage toxicity for every data points in all 9 plates. Because of the strong inter-well reproducibility in plates P1 to P4 (Figure S4) and to simplify our subsequent analysis, we first calculated the median of each variable for all replicates per plate in Exp1 & 2 before computing their SSMD* and comparing the results with the ones obtained in P5 to P9 (Figure S5). Pairwise comparison of all replicates within a plate (P1 to P4) confirmed data reproducibility (Figure S4 and Table 2). Performing inter-plate comparisons demonstrated also good reproducibility between plates prepared on the same day (compare P1 with P2 and P3 with P4) and between plates prepared on different days (Figure S5 and Table 3). We further classified all tested compounds in 5 phenotypic categories based on a threshold with at least 6 out of 9 replicates showing the same classified outcome: *Out* for compounds with no activity, inducing host macrophages cytotoxicity without leishmanicidal activity, or unclassifiable compounds exhibiting undefined phenotypes (1 case, C05, out of 60 tested compounds); *High* (threshold value of − 4.7), *Low* (threshold value between - 2 and - 4.7), and *Medium* for compounds that were classified as “High” and “Low” in independent replicate experiments for leishmanicidal compounds not toxic to the host macrophages; *∼Toxic* for leishmanicidal compounds that induce some toxicity on the host macrophages at this concentration level but would be worth validating at lower dosage. Based on this classification procedure, compounds tested with our HCA assay were categorized below in either irrelevant or relevant for the hit selection process:

**Table 2:** Pairwise comparison for replicates within P1 to P4.

**P1**	R1	R2	R3	R4	**P3**	R1	R2	R3	R4
R1		95	98	97	R1		100	100	97
R2			97	97	R2			100	97
R3				97	R3				97
R4					R4				
**P2**	R1	R2	R3	R4	**P4**	R1	R2	R3	R4
R1		100	97	95	R1		100	100	98
R2			97	93	R2			100	98
R3				92	R3				98
R4					R4				

The results for each replicate (R1 to R4) were compared and the rate of identical outcomes was reported after plate pairwise comparison.

**Table 3:** Pairwise comparison for all plate combinations.

	P1	P2	P3	P4	P5	P6	P7	P8	P9
**P1**		100 (100)	95 (93)	95 (93)	93 (90)	82 (75)	92 (85)	88 (82)	93 (87)
**P2**			95 (93)	95 (93)	93 (90)	82 (75)	92 (85)	88 (82)	93 (87)
**P3**				97 (92)	93 (90)	78 (72)	93 (87)	87 (80)	93 (87)
**P4**					93 (92)	82 (77)	93 (88)	90 (83)	93 (88)
**P5**						80 (77)	93 (90)	88 (82)	95 (92)
**P6**							82 (82)	88 (87)	82 (82)
**P7**								92 (88)	93 (92)
**P8**									90 (88)
**P9**									

The results were compared and the rate of similar (identical) outcomes was reported after plate pair concordance commutation. Of note, to compute the similarity rate we considered (*High* and *Low*) and (*C−* and *Cytotoxic*) as similar.

### Irrelevant compounds

**Figure 3:**
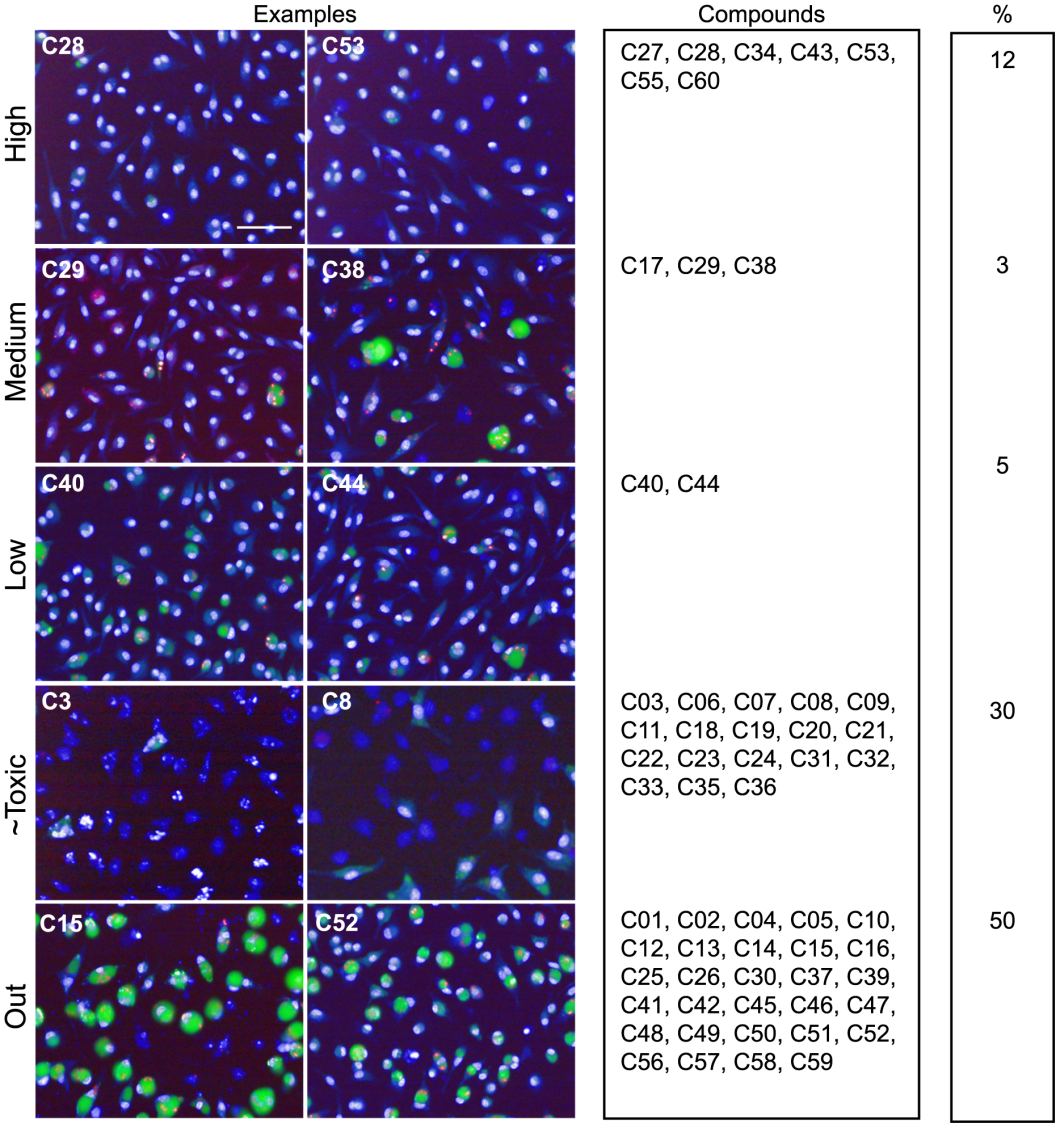
Screening results. Merged images for nucleus, PV and amastigote fluorescence of one acquisition field representing different phenotypes are displayed. Scale bar corresponds to 50 µm for all images. Each compound has been categorized into High, Low, ∼Toxic and Cytotoxic based on its SSMD values and following the decision tree described in Figure S3. The Medium class has been introduced to define compounds that were classified as “High” and “Low” in independent replicate experiments. Percentages per category are indicated.

**Figure 4:**
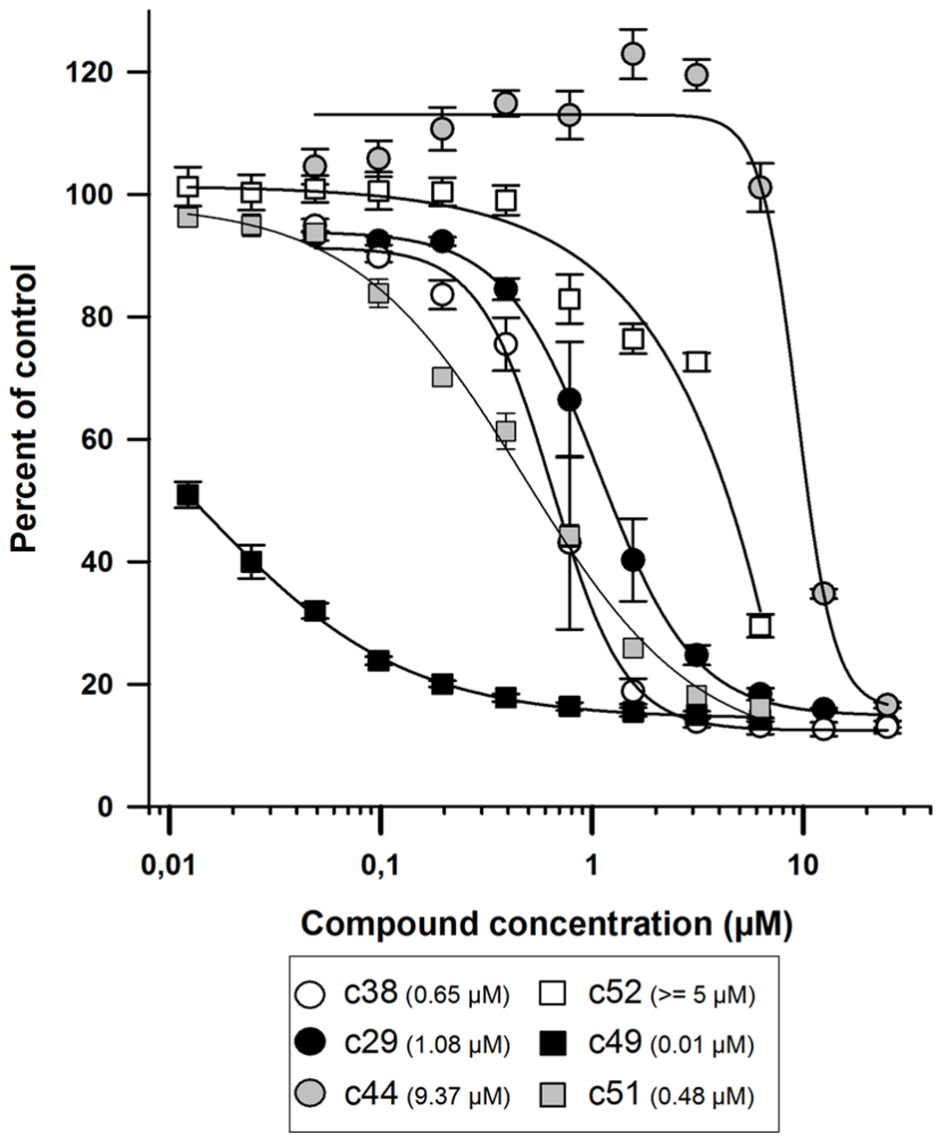
Effect of various anti-leishmanial molecules on the viability of *L. amazonensis* promastigotes. Procyclic promastigotes (2×10^4^/well) were incubated with different concentrations of compounds (0.0125 to 25 µM). Parasites cultured in medium alone (C−, 0% growth inhibition) or in the presence of 1 µM Amphotericin B (C+, 100% growth inhibition) were used as negative and positive controls, respectively. After 64 h incubation with compounds, resazurin (2.5 µg/ml) was added to each well and parasites were incubated for an additional 8 h before measuring resofurin fluorescence (λex = 550 nm, bandwidth = 9 nm; λem = 590 nm, bandwidth = 20 nm). Each compound concentration was tested in quadruplicates and the average ± standard deviation is displayed. EC50 values were calculated using SigmaPlot (SPSS, SSI, San Jose,CA, USA) 4-parameter logistic nonlinear regression analysis (values in parenthesis in the upper right panel).

Among the dominant class of 18 compounds that presented a strong anti-leishmanial activity associated with high toxicity to host macrophages a majority of compounds were Paullone derivatives (Figure 3, image C3 for an illustration). Paullones have been described previously as inhibitors of cyclin-dependent kinases and glycogen synthase kinase-3, and also as inhibitors of *L. donovani* axenic amastigotes [42]. High toxicity described against host cells for these molecules indicated that screening on host-free parasite populations can lead to false positive hits, a conclusion also reached by De Muylder and colleagues [18]. Some Paullone derivatives were reported as either inefficient within THP1 macrophages and/or too toxic for the host cell [42]. A second class of compounds (chalcone derivatives; compounds **c10**, **c13-16**) was identified that did not induce any noticeable phenotype in our assay (Figure 3), although they have been previously identified as active against *L. donovani* axenic amastigotes in the micromolar range [43]. Finally, Acivicin (**c49**), Aphidicolin (**c51**) and Phenyltoxamine (**c52**) did not exhibit any activity on intramacrophagic amastigotes (Figure 3), even though these molecules were described previously as potent growth inhibitors for *L. major* promastigotes by Sharlow and coworkers [10]. Lack of leishmanicidal activity of these compounds against intramacrophagic amastigotes was certainly due to their inability to cross the host macrophage membranes surrounding the parasites. Control experiments performed on *L. amazonensis* promastigotes indeed confirmed the activity of these compounds towards host-free parasites, with IC50 values of 0.01 µM, 0.48 µM, and > = 5 µM for Acivicin, Aphidicolin, and Phenyltoxamine, respectively (Figure 4).

### Relevant compounds

Anti-leishmanial compounds were distributed into two phenotypic categories. The first comprised 4 compounds with low leishmanicidal activity at 10 µM on intracellular amastigotes (**c29**, **c38**, **c40** and **c44**) (Figure 3). This category includes notably the known drugs Pentamidine Isethionate (**c38**), a molecule active on viscerotropic and dermotropic *Leishmania* species [44] different from *L. amazonensis*, Miconazole (**c40**) and Clotrimazole (**c44**), two antifungals with known anti-leishmanial activity [45], which showed variable efficacy depending on therapy conditions and *Leishmania* species [46]–[48]. Moreover, while Pentamidine Isethionate in particular has been recently validated as a strong growth inhibitor of *L. major* [10] for both promastigote and axenic amastigote–like stages with an EC50 value similar to the value we obtained with *L. amazonensis* promastigotes (0.65 µM, Figure 4), it only exhibited low level of intra-macrophage activity (above 10 µM EC50) [47]. Such discrepancies between hit compounds showing leishmanicidal activities against either promastigotes or intramacrophagic amastigotes have already been observed [18], [49], reinforcing the value of our approach.

The second category includes compounds that displayed a strong intra-macrophagic anti-leishmanial phenotype such as our reference compounds, Leu-oMe (**c43** and **c60**) and AmphoB (**c55**) and four compounds (**c28**, **c32**, **c34** and **c53**) (Figure 3) identified by Guiguemde and co-workers for their activity against *L. major* promastigotes [50].

Our results further reinforce the need and the adequacy of cellular assays such as the one presented here for rapid and successful identification of active molecules on *Leishmania* cell-cycling amastigotes hosted by primary macrophages [49].

### Concluding remarks

In the present study, we developed a scalable, throughput capable high content approach to select chemicals able to eliminate *L. amazonensis* amastigotes that are actively multiplying within the acidic giant parasitophorous vacuoles of primary macrophages.

This novel assay is simple and relies on only few experimental steps during which highly pure populations of amastigotes, compounds and fluorescent reporters are sequentially added to adherent macrophages without any washing or addition of fixative reagent. It generated robust and reproducible data based on the manipulation of large homogeneous populations of infected macrophages and allowed a dual measure of leishmanicidal activity against intramacrophagic parasites and the host macrophage health status.

By allowing real-time monitoring and kinetic studies on living adherent primary macrophages, this approach offers many advantages over assays that have been described recently in the literature with respect to assay reproducibility and infection homogeneity [11], [18], [49], [51]. Additionally, it enables the discovery of leishmanicidal compounds acting through the activation of microbicidal mechanisms of the host macrophage. Finally, the compound incubation period of our HCA assay was successfully prolonged up to six days (Figure S6) thus allowing for the discovery of slow-acting leishmanicidal compounds with kinetics similar to antimonials. Analyses of screening campaigns performed with compounds from kinase inhibitor libraries as part of the LeishDrug project sponsored by the EU's Seventh Framework Programme for Research are ongoing [52].

## Supporting Information

Figure S1 **Data classification decision tree.** Each computed SSMD* passed through successive decision points or steps (represented as diamonds indicating the parameters) to reach a compound classification (color-coded rounded rectangle indicating the class) based on their SSMD* values (green and red lines for no difference and significant difference to the DMSO control, respectively). Classes were defined as followed: **High** (green) and **Low** (yellow) for compounds with no toxic side effect on macrophages but strong or moderate leishmanicidal activities, respectively; **C−** (grey) for compounds with similar behavior as the negative DMSO control; **Cytotoxic** (black) for compounds inducing a strong toxicity on macrophages (significant SSMD* values for the VI, TM and HM parameters) and no leishmanicidal effect; **∼Toxic** (red) for compounds toxic for the host macrophages but still presenting strong leishmanicidal activity.
(TIF)

Figure S2 **FACS-based phenotype analysis of primary mouse macrophages before plating in 384-well plate.** Kinetics analysis of CD115 (**A**) and F4/80 (**B**) macrophage specific markers on adherent cells harvested at different time points during the culture of bone marrow cell in the presence of mrCSF-1. On day 6, the best yield of viable macrophage expressing high levels of CD115 and F4/80 was obtained (Thick solid line and data not shown). (**C, D**) Live macrophages recovered after 6 days were analyzed for the following markers: CD115, F4/80, CD11c, CD80 and CD11b, all of which are displayed at the plasma membrane of steady state mouse macrophages. The presence of MHC Class II molecules allows evaluating the percentage of non-steady state macrophages. The percentage of macrophages positive for these markers (**C**) and the Mean Fluorescence Intensity ratios (marker-specific over isotype control staining) (**D**) are shown. Box and whisker plots show the distribution of the data from 10 experiments: bottom line, 25th percentile; middle line, median; top line, 75th percentile; whiskers, fifth and 95th percentiles.
(TIF)

Figure S3 **1% DMSO does not induce toxicity to the macrophage population.** Boxplots with whiskers from minimum to maximum of number of viable cells for no treatment (“media”) versus 1% DMSO treated wells. Data were gathered from n = 15 and n = 64 wells for “media” and DMSO respectively on 4 independent 384-well plates. A 2-way ANNOVA test performed on each plate did not reveal significant differences between the two conditions.
(TIF)

Figure S4 **Fingerprint of hit classes after data classification for quadruplicate samples in plates P1 to P4.** All compounds are listed for every replicates (R1 to R4) in each plate (P1 to P4). The color code corresponds to the classes defined in Figure S3.
(PDF)

Figure S5 **Fingerprint of hit classes after data classification for all plates.** All compounds are listed for all plates (P1–P9). For clarity, the SSMD* of the median of the 4 replicates have been used for plates P1 to P4. P1/P2 and P3/P4 respectively have been performed in the same experiment (Exp1 and Exp2); all subsequent plates were performed in different experiments (Exp3 to Exp7) spread throughout 7 weeks. The color code corresponds to the classes defined in Figure S3.
(PDF)

Figure S6 **Control readouts after a 6-day incubation period.** Bi-parametric dot plots showing the robust SSMD values (SSMD*) of PV/HM and VI variables for control wells after a prolonged incubation period of 6 days. Controls for DMSO vehicle (C−, black diamonds, n = 24), leishmanicidal (Amphotericin B, C+, black circles, n = 20), and toxic (cycloheximide, C†, squares, n = 20) compounds are displayed. SSMD* calculations were performed using C− values for normalization as described in material and methods. These data indicate that up to 6 days of co-incubation does not alter the QC metrics of the HCA. Thus, our assay allows further characterization of low efficiency compounds that induce intermediate phenotype after a 3-days incubation period.
(TIF)

Table S1 **List of tested compounds.** Compounds with their characteristics, sources and references when available are listed.
(PDF)

Table S2 **Schematic representation of P1 to P4 plate design.** These plates contain compound quadruplicates and the 3 following controls: C− for 1% DMSO, C+ for 0.5 µM AmphoB and M for no addition.
(PDF)

Table S3 **Schematic representation of the P5 to P9 plate design.** A common plate design was used for P5 to P9 plates and contained one data point per compound and the following controls: (C−) for 1% DMSO, (C+) for 0.5 µM Amphotericin B and (C†) for 180 µM cycloheximide.
(PDF)

Table S4 **Theoretical values of the two quality control (QC) tests used (robust Z′Factor and SSMD) in the study.** We defined four categories (Excellent (E), Good (G), Acceptable (A) and Poor (P)) to qualify the QC metrics depending on the indicated threshold values.
(PDF)
