# Supplementary figures and images for: High Content Analysis of Primary Macrophages Hosting Proliferating Leishmania Amastigotes: Application to Anti-leishmanial Drug Discovery
Source: PLoS Negl Trop Dis. 2013 Apr 4;7(4):e2154. doi: 10.1371/journal.pntd.0002154 (PMC3617141; doi:10.1371/journal.pntd.0002154)

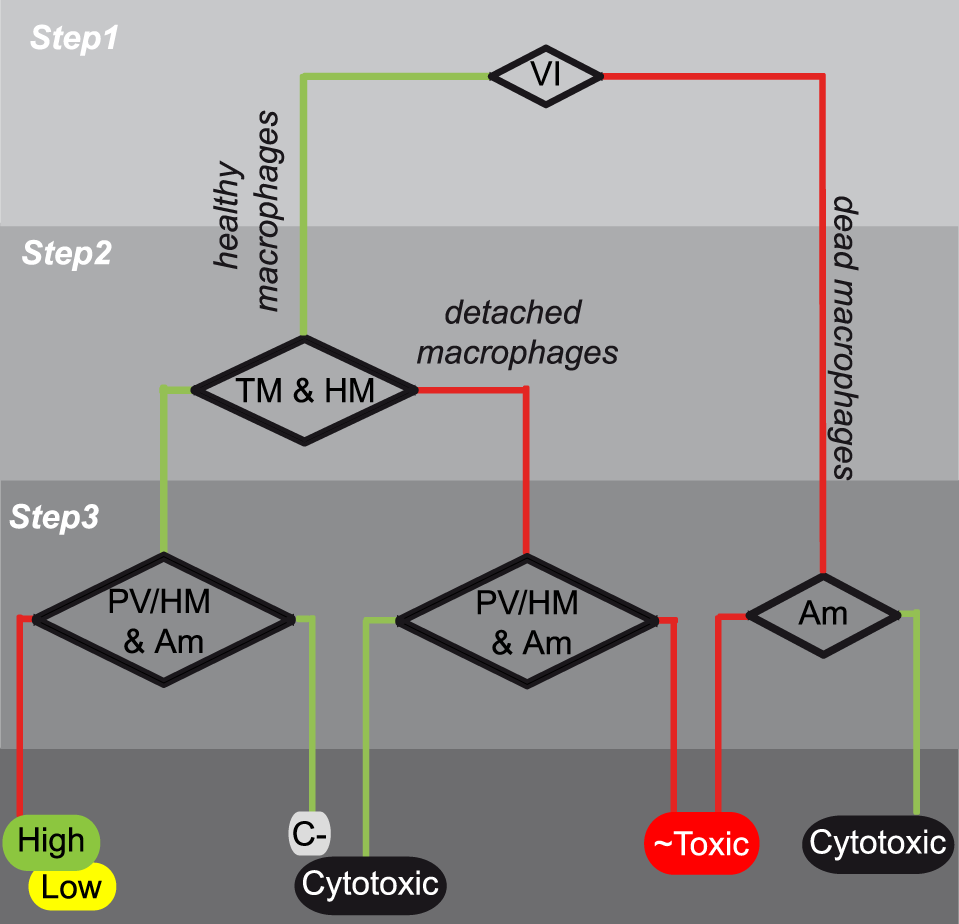

Supplement: Figure S1 — Data classification decision tree. Each computed SSMD* passed through successive decision points or steps (represented as diamonds indicating the parameters) to reach a compound classification (color-coded rounded rectangle indicating the class) based on their SSMD* values (green and red lines for no difference and significant difference to the DMSO control, respectively). Classes were defined as followed: High (green) and Low (yellow) for compounds with no toxic side effect on macrophages but strong or moderate leishmanicidal activities, respectively; C− (grey) for compounds with similar behavior as the negative DMSO control; Cytotoxic (black) for compounds inducing a strong toxicity on macrophages (significant SSMD* values for the VI, TM and HM parameters) and no leishmanicidal effect; ∼Toxic (red) for compounds toxic for the host macrophages but still presenting strong leishmanicidal activity. (TIF) [file pntd.0002154.s001.tif]

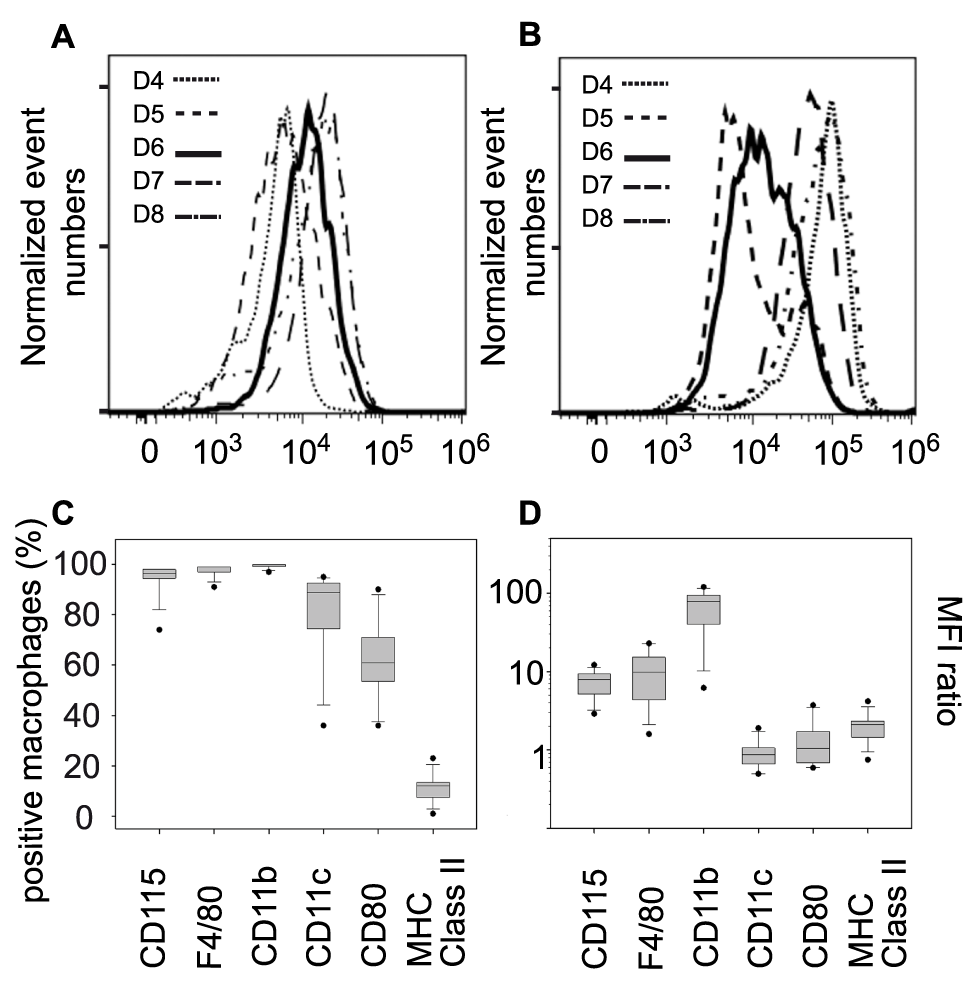

Supplement: Figure S2 — FACS-based phenotype analysis of primary mouse macrophages before plating in 384-well plate. Kinetics analysis of CD115 (A) and F4/80 (B) macrophage specific markers on adherent cells harvested at different time points during the culture of bone marrow cell in the presence of mrCSF-1. On day 6, the best yield of viable macrophage expressing high levels of CD115 and F4/80 was obtained (Thick solid line and data not shown). (C, D) Live macrophages recovered after 6 days were analyzed for the following markers: CD115, F4/80, CD11c, CD80 and CD11b, all of which are displayed at the plasma membrane of steady state mouse macrophages. The presence of MHC Class II molecules allows evaluating the percentage of non-steady state macrophages. The percentage of macrophages positive for these markers (C) and the Mean Fluorescence Intensity ratios (marker-specific over isotype control staining) (D) are shown. Box and whisker plots show the distribution of the data from 10 experiments: bottom line, 25th percentile; middle line, median; top line, 75th percentile; whiskers, fifth and 95th percentiles. (TIF) [file pntd.0002154.s002.tif]

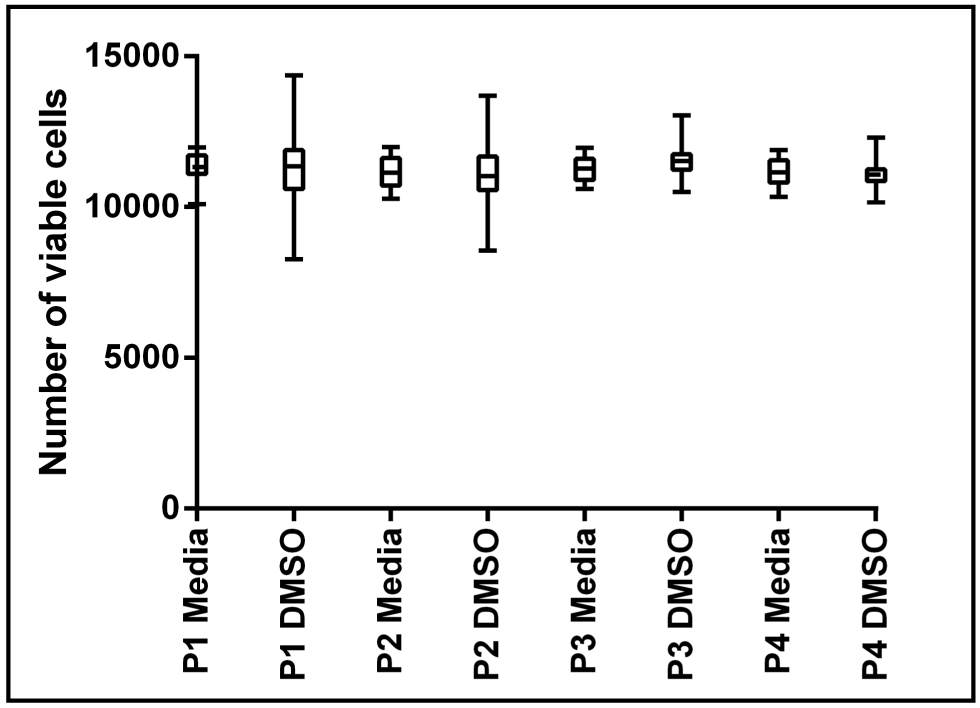

Supplement: Figure S3 — 1% DMSO does not induce toxicity to the macrophage population. Boxplots with whiskers from minimum to maximum of number of viable cells for no treatment (“media”) versus 1% DMSO treated wells. Data were gathered from n = 15 and n = 64 wells for “media” and DMSO respectively on 4 independent 384-well plates. A 2-way ANNOVA test performed on each plate did not reveal significant differences between the two conditions. (TIF) [file pntd.0002154.s003.tif]

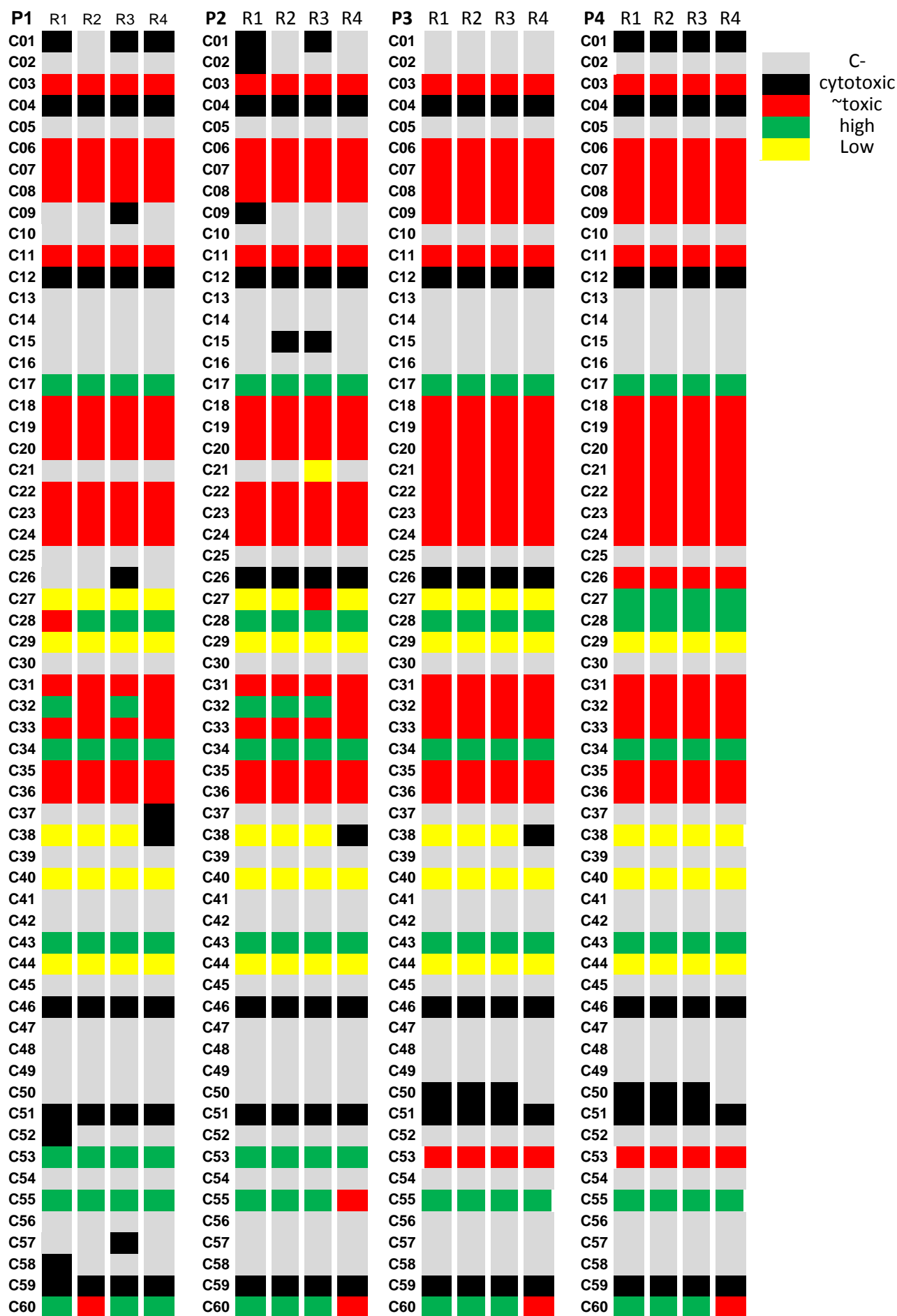

Supplement: Figure S4 — Fingerprint of hit classes after data classification for quadruplicate samples in plates P1 to P4. All compounds are listed for every replicates (R1 to R4) in each plate (P1 to P4). The color code corresponds to the classes defined in Figure S3. (PDF) [file pntd.0002154.s004.pdf]

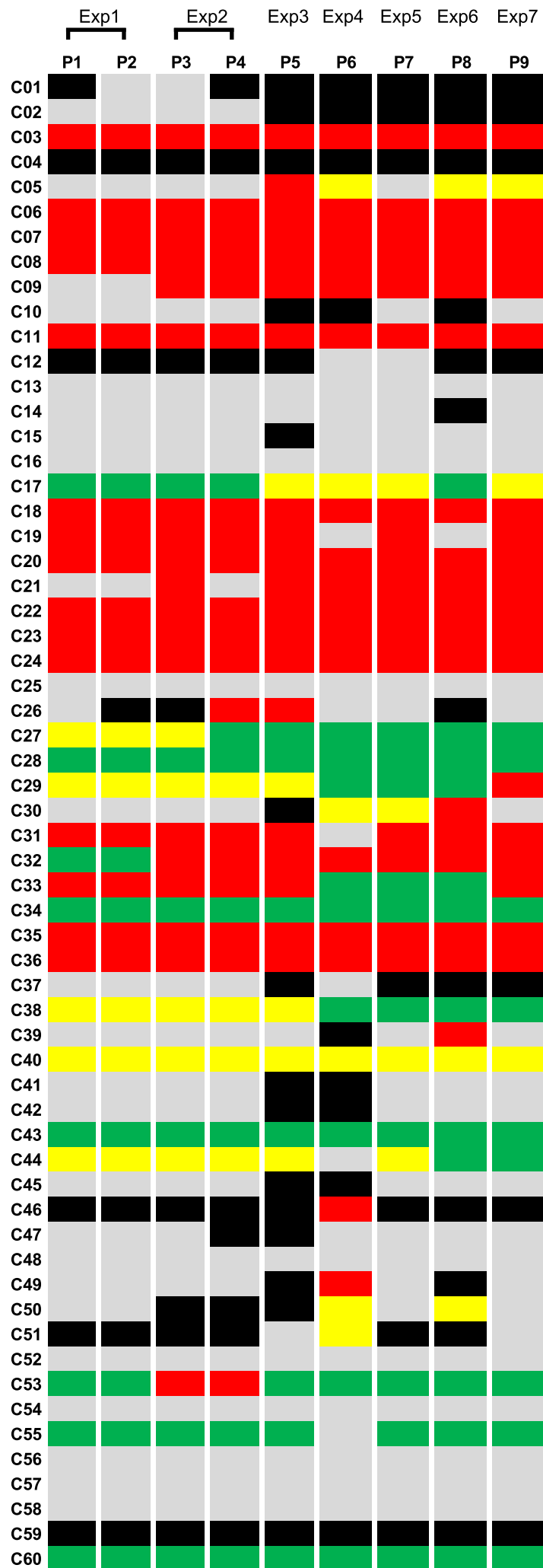

Supplement: Figure S5 — Fingerprint of hit classes after data classification for all plates. All compounds are listed for all plates (P1–P9). For clarity, the SSMD* of the median of the 4 replicates have been used for plates P1 to P4. P1/P2 and P3/P4 respectively have been performed in the same experiment (Exp1 and Exp2); all subsequent plates were performed in different experiments (Exp3 to Exp7) spread throughout 7 weeks. The color code corresponds to the classes defined in Figure S3. (PDF) [file pntd.0002154.s005.pdf]

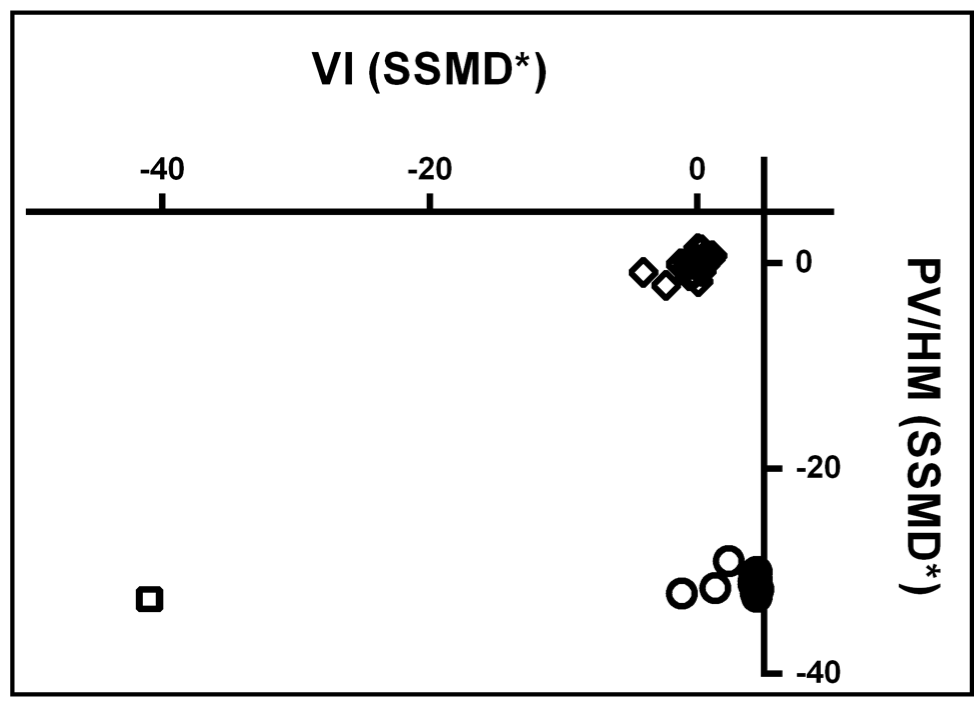

Supplement: Figure S6 — Control readouts after a 6-day incubation period. Bi-parametric dot plots showing the robust SSMD values (SSMD*) of PV/HM and VI variables for control wells after a prolonged incubation period of 6 days. Controls for DMSO vehicle (C−, black diamonds, n = 24), leishmanicidal (Amphotericin B, C+, black circles, n = 20), and toxic (cycloheximide, C†, squares, n = 20) compounds are displayed. SSMD* calculations were performed using C− values for normalization as described in material and methods. These data indicate that up to 6 days of co-incubation does not alter the QC metrics of the HCA. Thus, our assay allows further characterization of low efficiency compounds that induce intermediate phenotype after a 3-days incubation period. (TIF) [file pntd.0002154.s006.tif]
