# Supplementary material for: High Content Analysis of Primary Macrophages Hosting Proliferating Leishmania Amastigotes: Application to Anti-leishmanial Drug Discovery
Source: PLoS Negl Trop Dis. 2013 Apr 4;7(4):e2154. doi: 10.1371/journal.pntd.0002154 (PMC3617141; doi:10.1371/journal.pntd.0002154)
Supplement: Table S1 — List of tested compounds. Compounds with their characteristics, sources and references when available are listed. (PDF) [file pntd.0002154.s007.pdf]

compound number	Code/Name	MW (Da)	concentration (µM)	Source	Catalog Number	Published ID (if available)	Known Activity/Pharmacological Action	Reference
**1**	KuRei-24	330,38	10	Pr. Dr. Conrad Künick	NA	10b	kinase inhibitor (Paullone analog)	[1,2]
**2**	KuRei-28	350,80	10	Pr. Dr. Conrad Künick	NA	-	kinase inhibitor (Paullone analog)	
**3**	KuRei-38	372,46	10	Pr. Dr. Conrad Künick	NA	10e	kinase inhibitor (Paullone analog)	[1,2]
**4**	KuRei-68	341,36	10	Pr. Dr. Conrad Künick	NA	10d	kinase inhibitor (Paullone analog)	[1,2]
**5**	KuRei-101	334,34	10	Pr. Dr. Conrad Künick	NA	10c	kinase inhibitor (Paullone analog)	[1,2]
**6**	KuRei-131	316,35	10	Pr. Dr. Conrad Künick	NA	10a	kinase inhibitor (Paullone analog)	[1,2]
**7**	KuRei-147	440,56	10	Pr. Dr. Conrad Künick	NA	12f	kinase inhibitor (Paullone analog)	[1,2]
**8**	KuRei-148	468,97	10	Pr. Dr. Conrad Künick	NA	12e	kinase inhibitor (Paullone analog)	[1,2]
**9**	KuRei-152	464,55	10	Pr. Dr. Conrad Künick	NA	12d	kinase inhibitor (Paullone analog)	[1,2]
**10**	KuRei-153	327,38	10	Pr. Dr. Conrad Künick	NA	3a	anti-leishmanial (Chalcone derivative)	[1,2]
**11**	KuRei-169	434,53	10	Pr. Dr. Conrad Künick	NA	12c	kinase inhibitor (Paullone analog)	[1,2]
**12**	KuRei-170	355,43	10	Pr. Dr. Conrad Künick	NA	10f	kinase inhibitor (Paullone analog)	[1,2]
**13**	KuRei-218	333,41	10	Pr. Dr. Conrad Künick	NA	3d	anti-leishmanial (Chalcone derivative)	[1,2]
**14**	KuRei-219	317,34	10	Pr. Dr. Conrad Künick	NA	3e	anti-leishmanial (Chalcone derivative)	[1,2]
**15**	KuRei-223	328,36	10	Pr. Dr. Conrad Künick	NA	3f	anti-leishmanial (Chalcone derivative)	[1,2]
**16**	KuRei-256	250,29	10	Pr. Dr. Conrad Künick	NA	3i	anti-leishmanial (Chalcone derivative)	[1,2]
**17**	KuRei-289	346,38	10	Pr. Dr. Conrad Künick	NA	-		
**18**	KuRei-299	524,61	10	Pr. Dr. Conrad Künick	NA	12j	kinase inhibitor (Paullone analog)	[1,2]
**19**	KuRei-300	424,49	10	Pr. Dr. Conrad Künick	NA	12g	kinase inhibitor (Paullone analog)	[1,2]
**20**	KuRei-301	435,52	10	Pr. Dr. Conrad Künick	NA	12h	kinase inhibitor (Paullone analog)	[1,2]
**21**	KuRei-316	494,58	10	Pr. Dr. Conrad Künick	NA	12m	kinase inhibitor (Paullone analog)	[1,2]
**22**	KuRei-317	450,53	10	Pr. Dr. Conrad Künick	NA	12i	kinase inhibitor (Paullone analog)	[1,2]
**23**	KuRei-335	494,58	10	Pr. Dr. Conrad Künick	NA	12l	kinase inhibitor (Paullone analog)	[1,2]
**24**	KuRei-532	575,74	10	Pr. Dr. Conrad Künick	NA	-		
**25**	KuRei-557	507,58	10	Pr. Dr. Conrad Künick	NA	-		
**26**	KuRei-630	388,46	10	Pr. Dr. Conrad Künick	NA	-		
**27**	(Z)-2-((3-ethyl-5-methoxybenzo[d]thiazol-2(3H)-ylidene) methyl)-1-methylpyridinium	299,42	10	ChemDiv	4400-0074	SJ000027780		[3]
**28**	(E)-2-((1-ethyl-6-methylquinolin-2(1H)-ylidene)methyl)-1-methylpyridinium	277,39	10	ChemDiv	4400-0137	SJ000027785		[3]
**29**	(Z)-1-methyl-2-((3-methylbenzo[d]thiazol-2(3H)-ylidene)methyl) pyridinium	255,36	10	ChemDiv	4400-0141	SJ000027786		[3]
**30**	4-propyl-2,3,5,6,7,8-hexahydro-1H-cyclopenta[b] quinolin-9(4H)-imine	230,36	10	ChemDiv	4817-2829	SJ000031505		[3]
**31**	(E)-2-(4-(dimethylamino)styryl)-1-ethyl-6-methoxyquinolinium	333,46	10	ChemDiv	6253-0474	SJ000112968		[3]
**32**	(E)-2-(4-(diethylamino)styryl)-1-ethyl-7-methoxyquinolinium	361,51	10	ChemDiv	6253-0494	SJ000112969		[3]

compound number	Code/Name	MW (Da)	concentration (µM)	Source	Catalog Number	Published ID (if available)	Known Activity/Pharmacological Action	Reference
**33**	(Z)-2-((3-(2-hydroxyethyl)-6-methylbenzo[d]thiazol-2(3H) -ylidene)methyl)-3-methylbenzo[d]thiazol-3-ium	355,50	10	ChemDiv	7165-0022	SJ000044413		[3]
**34**	(E)-2-((1-allylpyridin-2(1H)-ylidene)methyl)-3-methylbenzo[d] thiazol-3-ium	281,40	10	ChemDiv	7771-0711	SJ000045729		[3]
**35**	(E)-2-((1,6-dimethylquinolin-2(1H)-ylidene)methyl)-3-methylbenzo[d] thiazol-3-ium	319,45	10	ChemDiv	7771-0743	SJ000045732		[3]
**36**	3-allyl-2-((1-ethylpyridin-4(1H)-ylidene)methyl)benzo[d] thiazol-3-ium	295,43	10	ChemDiv	7771-0802	SJ000045737		[3]
**37**	Wortmannin	428,40	10	Aldrich	W1628		PI3 Kinase inhibitor, pro apoptotic	[4]
**38**	Pentamidine isethionate salt	592,68	10	Aldrich	439843		anti-microbial, anti-fungal, anti-protozoal, phosphatase inhibitor	[5,6]
**39**	Paromomycin sulfate salt	713,71	10	Aldrich	P5057		anti-microbial	
**40**	(+/-)-Miconazole nitrate salt	479,14	10	Aldrich	M3512		anti-fungal	[7]
**41**	Curcumin	368,39	10	Aldrich	C7727		anti-tumor, pro-apoptotic, anti-inflammatory, anti-oxydant	
**42**	Allopurinol	136,11	10	Aldrich	A8003		Inhibitor of xanthine oxidase and *de novo* pyrimidine biosynthesis	
**43**	L-Leucine methyl ester hydrochloride	181,66	1000	Aldrich	L9000		anti-leishmanial	
**44**	Clotrimazole	344,84	10	Aldrich	C6019		anti-fungal, local anti-infective	[6,7]
**45**	Fluconazole	306,27	10	Aldrich	F8929		anti-fungal	
**46**	Itraconazole	705,63	10	Aldrich	I6657		broad spectrum anti-fungal	
**47**	Edelfosine	523,73	10	Aldrich	O-1415		anti-neoplastic	
**48**	Ilmofosine	525,77	10	Aldrich	I2409		PKC Inhibitor	
**49**	Acivicin	178,57	10	Aldrich	A2295		anti-neoplastic, antibiotic, anti-fungal enzyme inhibitor	[6]
**50**	Tetraethylthiuram disulfide	296,54	10	Aldrich	86720		Alcohol dehydrogenase inhibitor.	
**51**	Aphidicolin from Nigrospora sphaerica	338,48	10	Aldrich	A0781		antibiotic, potent anti-viral, anti-mitotic agent and DNA polymerase inhibitor	[6]
**52**	Phenyltoloxamine citrate salt	447,48	10	Aldrich	P8404		anti-histamine	[6]
**53**	(E)-2-((1-allylpyridin-2(1H)-ylidene)methyl) -3-ethylbenzo[d]thiazol-3-ium	295,43	10	ChemDiv	7771-0817	SJ000045738		[3]
**54**	Pamidronate disodium salt hydrate	369,10	10	Aldrich	P2371		anti-osteoclastic, anti- protozoa	[8]
**55**	Amphotericin B	924,08	0,5	Merck	171375		anti-fungal antibiotic, anti-leishmanial	
**56**	Ketoconazole	531,43	10	Merck	420600		anti-fungal	
**57**	Sodium stibogluconate	907,90	10	Merck	567565		pentavalent antimonial known anti-leishmanial	
**58**	Allopurinol riboside	268,23	10	Aldrich	A2026		anti-leishmanial	
**59**	Miltefosine	407,00	10	Merck	475841		pro-apoptotic	
**60**	L-Leucine methyl ester hydrochloride	181,66	1000	Aldrich	61890		anti-neoplastic, anti-protozoal	

1.Reichwald C, Shimony O, Dunkel U, Sacerdoti-Sierra N, Jaffe CL, et al. (2008). J Med Chem 51: 659-665.
2. Reichwald C, Shimony O, Sacerdoti-Sierra N, Jaffe CL, Kunick C (2008) . Bioorganic & medicinal chemistry letters 18: 1985-1989.
3. Guiguemde WA, Shelat AA, Bouck D, Duffy S, Crowther GJ, et al. (2010) Chemical genetics of *Plasmodium falciparum* . Nature 465: 311-315.
4. Wymann MP, Bulgarelli-Leva G, Zvelebil MJ, Pirola L, Vanhaesebroeck B, et al. (1996). Mol Cell Biol 16: 1722-1733.
5. Azas N, Di Giorgio C, Delmas F, Gasquet M, Timon-David P (1997). Exp Parasitol 87: 1-7.
6. Sharlow ER, Close D, Shun T, Leimgruber S, Reed R, et al. (2009). PLoS Negl Trop Dis 3: e540.
7. Berman JD (1981). Am J Tropl Med Hyg 30: 566-569.
8. Yardley V, Khan AA, Martin MB, Slifer TR, Araujo FG, et al. (2002). Antimicrob Agents Chemother 46: 929-931.
