# Supplementary material for: High Content Analysis of Primary Macrophages Hosting Proliferating Leishmania Amastigotes: Application to Anti-leishmanial Drug Discovery
Source: PLoS Negl Trop Dis. 2013 Apr 4;7(4):e2154. doi: 10.1371/journal.pntd.0002154 (PMC3617141; doi:10.1371/journal.pntd.0002154)
Supplement: Table S4 — Theoretical values of the two quality control (QC) tests used (robust Z′Factor and SSMD) in the study. We defined four categories (Excellent (E), Good (G), Acceptable (A) and Poor (P)) to qualify the QC metrics depending on the indicated threshold values. (PDF) [file pntd.0002154.s010.pdf]

**Z' Factor**	**SSMD[+]**	**QC Interpretation**
Z'≥0.5	SSMD≤–6.67	Excellent (**E**)
0.25≤Z'<0.5	–6.67<SSMD≤–4.7	Good (**G**)
0≤Z'<0.25	–4.7<SSMD≤–3	Acceptable (**A**)
Z'<0	SSMD>–3	Poor (**P**)

(+) from Zhang XD, 2008
